# Choose Your Label Wisely: Water-Soluble Fluorophores Often Interact with Lipid Bilayers

**DOI:** 10.1371/journal.pone.0087649

**Published:** 2014-02-04

**Authors:** Laura D. Hughes, Robert J. Rawle, Steven G. Boxer

**Affiliations:** Department of Chemistry, Stanford University, Stanford, California, United States; University of Geneva, Switzerland

## Abstract

Water-soluble organic fluorophores are widely used as labels in biological systems. However, in many cases these fluorophores can interact strongly with lipid bilayers, influencing the interaction of the target with the bilayer and/or leading to misleading fluorescent signals. Here, we quantify the interaction of 32 common water-soluble dyes with model lipid bilayers to serve as an additional criterion when selecting a dye label.

## Introduction

Organic fluorophores are ubiquitous tags for a host of biological targets, including cytosolic proteins, membrane proteins, and antibodies. Many of these targets are studied in systems that contain model or cellular lipid membranes. In our work studying model lipid bilayers, we have noticed that some water-soluble fluorescent dyes interact strongly with lipid membranes, even when the dyes are charged. This interaction of dyes with lipid bilayers can be the source of two general problems. First, dyes conjugated to biological targets can alter the target’s interaction with lipid bilayers, possibly even pulling them into the membrane, and may also change the interaction between targets themselves. Second, if free fluorophores remain in the sample after the labeling procedure, they can interact with lipid membranes and yield false signals, being mistakenly identified as membrane-bound targets.

Based on discussions with investigators from many labs, problematic dye-membrane interactions are widely noticed. For instance, two recent reports have measured how different fluorophores stick to microscope substrates [1] and how they can alter protein diffusion [2], highlighting the prevalence of these problems in all imaging contexts. In general, however, researchers tend to rely on anecdotal experience when choosing a fluorophore rather than objective criteria. Moreover, if dye manufacturers provide any description of the hydrophilicity of a probe, they only use qualitative terms such as “moderately hydrophilic.” Measurements have been made to quantify peptide- or drug-membrane interactions [3]–[6], but to our knowledge no systematic measurements have been made on commercially available fluorophores with lipid bilayers. We decided, therefore, to make systematic, quantitative measurements of the interaction of common water-soluble dyes with model lipid bilayers to guide the selection of fluorescent probes.

## Materials and Methods

### Materials

Alexa 488 carboxylic acid/succinimidyl ester, Alexa 532 carboxylic acid/succinimidyl ester, Alexa 532 C_5_ maleimide, Alexa 546 carboxylic acid/succinimidyl ester, Alexa 555 C_2_ maleimide, Alexa 568 hydrazide, Alexa 594 C_5_ maleimide, Alexa 633 C_5_ maleimide, Alexa 647 carboxylic acid/succinimidyl ester, Alexa 647 C_2_ maleimide, BODIPY-TMR C_5_ maleimide, 5,6-carboxyfluorescein, Oregon Green 488 maleimide (OG 488), Oregon Green 514 carboxylic acid/succinimidyl ester (OG 514), sulforhodamine B, Texas Red C_2_ maleimide, and tetramethylrhodamine-5-maleimide (TMR) were all obtained from Molecular Probes (Invitrogen, Eugene, OR). Atto 465 NHS-ester, Atto 488 NHS-ester, Atto 532 NHS-ester, Atto 550 maleimide, Atto 565 biotin, Atto 647 NHS-ester, Atto 647N maleimide, and Atto 655 NHS-ester were all obtained from ATTO-TEC (Siegen, Germany). Abberior STAR 635P azide was obtained from Abberior (Göttingen, Germany). Chromeo 488 NHS-ester was obtained from Active Motif (Carlsbad, CA). Dyomics 654 NHS-ester was obtained from Dyomics (Jena, Germany). Cy3 NHS-ester was kindly provided by Professor Justin Du Bois (Stanford University). Cy3B NHS ester, Sulfo-Cy3 maleimide and Sulfo-Cy5 maleimide were obtained from Amersham (GE Healthcare Biosciences, Pittsburgh, PA). Note that the molecules we have named Sulfo-Cy3 and Sulfo-Cy5 are also often referred to as Cy3 or Cy5 elsewhere (the nomenclature is inconsistent). The structures for the dyes (when available) are shown in Figure S1. Egg phosphatidylcholine (Egg PC) and dioleoyl phosphatidylserine (DOPS) were purchased from Avanti Polar Lipids (Alabaster, AL).

### Dye Solution Preparation

To prepare the dye stock solutions, 10 µl of 7–20 mM dye in anhydrous DMSO was diluted into 500 µl of PBS buffer (Gibco; 155.17 mM NaCl, 1.06 mM potassium phosphate monobasic, 2.97 mM sodium phosphate dibasic, pH 7.4). Stock solutions of dyes were incubated at room temperature for 2 h to mimic typical labeling reaction conditions, and then stored at −20°C in the dark until use. Immediately before use, stock solutions in PBS were thawed, and the absorbance at the dye’s maximum was measured using a Nanodrop (Thermo Fisher, Wilmington, DE). The concentration of dye was calculated from the published extinction coefficient (listed in Table 1), and a 400 nM solution in PBS was made. Immediately before addition to the dialysis membrane, the dye solution was sonicated in a bath sonicator for 30 s to disrupt any dye aggregates.

**Table 1:** MIF Values, Calculated Log D Values, and Photophysical Characteristics of Common Water-Soluble Dyes.

Dye^a^	MIF_corr_ b	logD_hyd_ c	logD_unhyd_ c	λ_max_(ex)	λ_max_(em)	ε (M^−1^ cm^−1^)^d^	QY^d^	DataSource^e^
Abberior STAR 635P azide	0.21±0.02	n/a	0.58	634	654	80000	0.55	[9], [10]
Alexa 488 SE	−0.003±0.007	−11.09	−8.02	494	517	73000	0.92	[11], [12]
Alexa 532 SE	0.04±0.01	−3.26	−0.16	530	555	81000	0.61	[11], [12]
Alexa 532 M*	0.58±0.05	−3.61	0.13	528	552	78000	0.61	[12], [13]
Alexa 546 SE	0.18±0.03	−3.68	−1.43	554	570	112000	0.79	[11], [12]
Alexa 555 M	0.04±0.03	–	–	556	572	158000	0.1	[12], [13]
Alexa 568 hydrazide	0.04±0.01	n/a	−5.89	576	599	86000	0.69	[12], [14]
Alexa 594 M	0.3±0.1	−7.4	−3.66	588	612	96000	0.66	[12], [13]
Alexa 633 M	8.0±0.5	−3.44	0.3	622	640	143000	–	[13], [15]
Alexa 647 SE	0.03±0.02	−6.72	−3.72	651	672	270000	0.33	[11], [12], [16]
Alexa 647 M	0.04±0.02	−8.1	−4.26	651	671	265000	0.33	[12], [13], [16]
Atto 465 SE	0.234±0.008	−1.12	−2.52	453	508	75000	0.75	[17]
Atto 488 SE	0.007±0.004	−7.6	−4.67	501	523	90000	0.8	[17]
Atto 532 SE	0.03±0.02	−6.48	−3.58	532	553	115000	0.9	[17]
Atto 550 M**	33±3	2.67	6.41	554	576	120000	0.8	[17], [18]
Atto 565 biotin	0.7±0.1	n/a	3.35	563	592	120000	0.9	[17]
Atto 647 SE**	0.87±0.03	–	–	645	669	120000	0.2	[17]
Atto 647N M	13±1	3.82	3.26	644	669	150000	0.65	[17], [19]
Atto 655 SE	0.15±0.03	−0.61	1.44	663	684	125000	0.3	[17], [20]
BODIPY-TMR M	93±8	−1.51	−1.96	544	570	60000	–	[13]
Carboxyfluorescein	0.02±0.01	n/a	−5.29	492	515	81000	–	[21]
Chromeo 488 SE	0.06±0.02	–	–	488	517	73000	–	[22]
Cy3 SE	7.8±0.4	4.69	3.29	555	570	150000	0.31	[23]
sulfo-Cy3 M	0.28±0.04	−2.72	1.12	550	570	150000	0.15	[24]
Cy3B SE	0.13±0.04	−0.62	2.38	559	570	130000	0.7	[25]
sulfo-Cy5 M	0.31±0.03	−2.19	1.65	649	670	250000	0.28	[26]
Dyomics 654 SE	0.10±0.02	−11.95	−9.03	653	677	220000	–	[27]
OG 488 M	0.04±0.01	−5.71	−2.35	496	524	81000	–	[13]
OG 514 SE	0.02±0.01	−4.92	−2.04	506	526	85000	–	[21]
Sulforhodamine B	2.0±0.1	n/a	−0.08	565	586	84000	–	[14]
Texas Red M	2.7±0.2	−3.19	2.87	595	615	112000	–	[13]
TMR M	0.35±0.02	−0.88	0.29	541	567	91000	–	[13]

a Reactive groups include maleimides (M), azide, biotin, hydrazide, and succinimidyl esters (SE). Where available, dye structures are given in Figure S1.

b Calculated using Equation 3. Error values are the propagated error from the standard deviation of three separate measurements each of the experimental and control samples.

c For dyes with hydrolysable reactive groups (i.e. Maleimide or Succinimidyl Esters), logD_hyd_ is the calculated log D using the molecular structure of the dye with a hydrolyzed reactive group. logD_unhyd_ is the calculated log D using the structure of the unhydrolyzed reactive group. For dyes without hydrolysable reactive groups, logD_unhyd_ is the calculated log D. For dyes without a calculated log D value, chemical structures were not available.

d **ε is the extinction coefficient at λ_max_ (ex). QY is the quantum yield. Both are listed as reported by the manufacturers in data sources.**

e Sources of the dye structures in Figure S1 and the photophysical data (λ_max_ (ex), λ_max_ (em), extinction coefficient, and quantum yield) reported in this table, as listed in the references.

* Indicates that two separate measurements instead of three were averaged for the experimental samples.

** Indicates that two separate measurements instead of three were averaged for the control samples.

### Vesicle Preparation

To form vesicles, Egg PC in chloroform or a 9∶1 molar mixture of EggPC:DOPS in chloroform was dried under a stream of nitrogen followed by desiccation under house vacuum for at least 2 h. The lipid film was rehydrated in PBS to a nominal concentration of 40 mg/ml and extruded through a 50 nm polycarbonate filter (Avanti Polar Lipids). This procedure yields unilamellar vesicles whose average hydrodynamic diameter is approximately 70 nm, as determined by dynamic light scattering measurements on similarly prepared vesicles (data not shown).

### Vesicle-Dye Dialysis

Dialysis was performed using 10 kD MWCO microdialysis cassettes (Pierce, Thermo Scientific, Rockford, IL). Vesicles in PBS were mixed 1∶1 with 400 nM dye solution to yield a final dye concentration of 200 nM and final lipid concentration of 20 mg/mL, and 100 µl of this vesicle-dye mixture was loaded into a prehydrated cassette. The dialysis cassette was then immersed in 1.4 ml of 200 nM dye solution in PBS inside a microcentrifuge tube, and the top was sealed with Parafilm. Note this procedure starts with the dye being approximately equilibrated across the dialysis membrane, to decrease the time to achieve equilibration of the dye-bilayer interaction.

To account for any differential loss of dye or aggregation on either side of the membrane, a control sample was run with 100 µl of 200 nM dye loaded into the dialysis cassette instead of the vesicle-dye mixture. For background measurements, the dialysis cassette was loaded with 100 µL of 20 mg/mL vesicles and immersed in 1.4 mL of PBS buffer (no dye present on either side of the dialysis membrane). For all samples, dialysis was allowed to proceed at room temperature in the dark for 3 days (68–76 h) on a platform shaker. All samples were prepared in triplicate. Note that for BODIPY-TMR, substantial adsorption of the dye to all tubing and pipette tips prevented an accurate measurement of the initial dye concentration.

### Fluorescence Measurements of Membrane-dye Association

**Figure 1:**
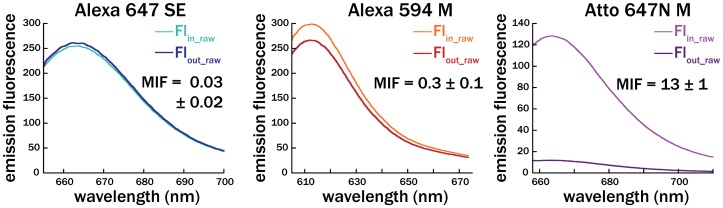
Example raw fluorescence spectra of three dyes. The dyes are representative of low (Alexa 647-SE), moderate (Alexa 594-M), and high (Atto 647N-M) MIF values. Fl_in_raw_ is the raw fluorescence spectrum of the vesicle solution inside the dialysis cassette, collected and prepared as described above. Fl_out_raw_ is the raw fluorescence spectrum of the solution outside the dialysis cassette. MIF values are the corrected MIF values averaged across three separate measurements, as reported in Table 1. Emission fluorescence is in arbitrary units.

For each sample, the contents of the dialysis membrane were removed after 3 days. 95 µl of the solution inside the dialysis membrane or 95 µl of the solution outside the dialysis membrane was mixed with 1.905 ml of 1% Triton-X in PBS in a poly (methyl methacrylate) cuvette (VWR, Radnor, PA). Mixtures were pipetted to mix, and the emission spectra were measured on a fluorimeter (Perkin Elmer LS 55, Waltham, MA). Slit widths on the monochromator were adjusted for each dye to obtain high signal without saturating the detector, and the same fluorimeter settings were used for all measurements of a given dye. Example fluorescence spectra of solutions inside and outside the dialysis cassette for dyes with different MIF values are shown in Figure 1 below. Raw fluorescence spectra data for all measurements on all dyes is provided in File S1.

Three spectra were averaged for each sample, and the averaged emission spectrum was integrated and then corrected using the appropriate background sample (either pure PBS or vesicle solution inside the dialysis membrane that was never exposed to dye). To account for the exclusion volume of the vesicles, which is inaccessible to dye molecules that cannot transverse the lipid bilayer, the integrated fluorescence of the solution inside the dialysis cassette was multiplied by the exclusion volume factor (1.06), calculated using the approximate average vesicle diameter (70 nm) and the nominal lipid concentration in the dialysis cassette (20 mg/mL). While the assumption that the dye is inaccessible to the interior of the vesicles will not hold for dyes which intercalate into the lipid bilayer, this correction is intended to create a conservative estimate for the MIF value. This corrected fluorescence value is referred to as *Fl_in_* in the section below.

### Calculation of Membrane Interaction Factor (MIF)

We define the MIF value as the ratio of dye fluorescence associated with the vesicles, 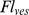, to dye fluorescence in the buffer, 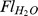, as shown in Eq. 1.
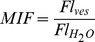
(1)



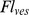
 cannot be directly measured, but can be obtained as the difference between the fluorescence inside the dialysis cassette (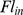) and outside (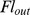), as shown in Eq. 2. 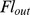 is equivalent to 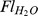.
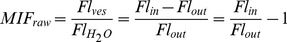
(2)


To correct for incomplete equilibration across the dialysis membrane due to dye aggregation or other mechanisms, the ratio of 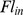 and 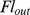 is normalized to the same ratio for the control sample (see Eq. 3), which is prepared in an identical fashion to the experimental sample, but without vesicles (see methods above). In this case, 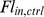 and 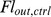 are the background subtracted fluorescence for the control sample inside and outside the dialysis cassette, respectively.
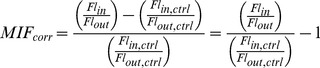
(3)


For most dyes, this correction is not needed since 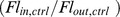 is approximately 1. However, for some dyes (most notably many with higher MIF values), this correction increased *MIF_raw_* by 10 or 20%. We assume this is the result of dyes with higher MIF values having a higher propensity to aggregate in solution. At a qualitative level, this assumption was supported by observing aggregates via fluorescence microscopy for a subset of these dyes (data not shown). Since there is a larger volume outside the dialysis membrane, aggregation would preferentially decrease the external concentration of dye able to cross the dialysis membrane and would therefore decrease the observed MIF value. Consequently, in Tables 1 and 2, we report *MIF_corr_*, the higher and more conservative estimate of dye association; the *MIF_raw_* values are reported in Tables S1 and S2 in File S1.

**Table 2:** Comparison of MIF Values Measured Against Zwitterionic 100% Egg PC Vesicles (MIF_corr_) and Against Negatively Charged 90% Egg PC/10% DOPS Vesicles (MIF_corr_neg_) for a Subset of Dyes.

Dye^a^	MIF_corr_ b	MIF_corr_neg_ c
Alexa 546 SE	0.18±0.03	0.04±0.07
Alexa 633 M	8.0±0.5	3.6±0.3
Atto 550 M	33±3	39±5
Atto 647N M	13±1	19±2
sulfo-Cy5 M	0.31±0.03	0.16±0.03
TMR M	0.35±0.02	0.18±0.04

a Reactive groups include maleimides (M) and succinimidyl esters (SE). Where available, dye structures are given in Figure S1.

b Corrected MIF value, measured using zwitterionic vesicles (100% Egg PC), and calculated using Equation 3. Error values are the propagated error from the standard deviation of three separate measurements each of the experimental and control samples. These are the same values as in Table 1.

c Corrected MIF value, measured using negatively charged vesicles (90% Egg PC, 10% DOPS), and calculated using Equation 3. Error values are the propagated error from the standard deviation of three separate measurements each of the experimental and control samples.

### Log D Calculations

Log D values at pH 7.4 were calculated in MarvinSketch v. 5.12.3 (Chem Axon, Cambridge, MA), based on the structures in Figure S1. Cl^−^ concentration was held at 155 mM and Na^+^ and K^+^ concentration at 162 mM, the log P values were equally weighted by three methods (VG, KLOP, and PHYS), and tautomerization and resonance were considered.

### Epifluorescence Measurements of Dye-Supported Bilayer (SLB) Interactions

Egg PC glass-supported bilayers were prepared as described previously [7], but using a different buffer. Briefly, Egg PC vesicles at 0.4 mg/mL in 10 mM NaH_2_PO_4_, 240 mM NaCl pH 7.4 buffer were pipetted into 40 µL Coverwell perfusion gaskets (Invitrogen) adhered to plasma-cleaned glass coverslips and were then incubated at room temperature for 20 min. This procedure allows the vesicles to adsorb to the glass surface at high density and rupture to form a continuous lipid bilayer. Following incubation, the supported bilayer was rinsed extensively with PBS (>5 mL).

Dye solutions, prepared in PBS at either 1 µM (for microscopy images) or at 20 nM (Movie S1), were briefly vortexed and immersed for 30 sec in a bath sonicator to disrupt any aggregates. 40 µL of dye solution was then pipetted into the gasket above the SLB and mixed thoroughly by pipetting back and forth. This procedure results in an approximately 2× dilution of the dye solution. After incubating for 5 min at room temperature, the gasket was thoroughly rinsed with PBS (>5 mL) to remove any dye solution. The supported bilayer was then imaged by epifluorescence to visualize any dye molecules or aggregates which remained associated with the bilayer. At least ten different regions on the supported bilayer were imaged. Note that this procedure is designed to visualize dyes which have longer-lasting interactions with the bilayer; dye molecules which only transiently interact with the bilayer will not be observed as they will be washed away during the extensive rinsing.

To properly compare image intensities between different dyes, it is necessary to account for the different imaging conditions which were used for each dye (e.g. different excitation/emission filters, quantum efficiency, etc.). Therefore, we normalized each supported bilayer image to the average intensity of a 500 nM dye solution pipetted onto a glass coverslip, imaged using the same objective and excitation/emission filters as were used to image the supported bilayers. All microscopy images shown are these normalized images.

### Microscopy

All epifluorescence micrographs were obtained using a Nikon Ti-U microscope with a 100x oil immersion objective, NA = 1.49 (Nikon Instruments, Melville, NY). For all images, the excitation source was a Spectra-X (Lumencor, Beaverton, OR). For Movie S1, the excitation source was a 633 nm He-Ne laser (75 mW, Melles Griot, Carlsbad, CA). Images were recorded using an Andor iXon 897 EMCCD camera (Andor Technologies, Belfast, UK), and were captured with Metamorph software (Molecular Devices, Sunnyvale, CA).

## Results and Discussion

We measured the interaction of 32 water-soluble fluorophores with unilamellar lipid vesicles using dialysis. In most cases, we measured reactive dyes, conjugated to a succinimidyl ester, maleimide, etc. Before exposure to lipid vesicles, dyes were dissolved in buffer for 2 hours at room temperature to mimic reaction conditions commonly used to attach dyes to biological targets. Then, a portion of dye solution was mixed with Egg PC vesicles, added to a 10 kD dialysis cassette, and dialyzed against the remaining dye solution. After three days, the solutions were removed from inside and outside the dialysis cassette, and the fluorescence of these solutions was measured on a fluorimeter (example spectra in Figure 1). After accounting for aggregation, quantum yield changes, and the exclusion volume of the vesicles (see Materials and Methods), we calculated the ratio of the fluorescence associated with the vesicles to the fluorescence in the aqueous solution [5], [6]. We call this ratio the membrane interaction factor (MIF), which represents the extent of interaction of the dye with the lipid vesicles. The MIF is related to the equilibrium partition coefficient, and in many cases may be the same within a proportionality factor which is dependent on the lipid concentration (see File S1). However, as an equilibration time course was not measured for every dye, we cannot claim that the MIF is necessarily proportional to the true equilibrium partition coefficient.

The MIF should be near zero for dyes that do not interact with the vesicles, and will be higher for those that interact more strongly. We note that the MIF value only provides a measure of the extent of interaction and gives no insight into the mechanism or kinetics of interaction. Possible mechanisms include binding to chemical groups on the vesicle surface, insertion into the bilayer at the head group/hydrophobic core interface, and long lasting intercalation into the hydrophobic core.

**Figure 2:**
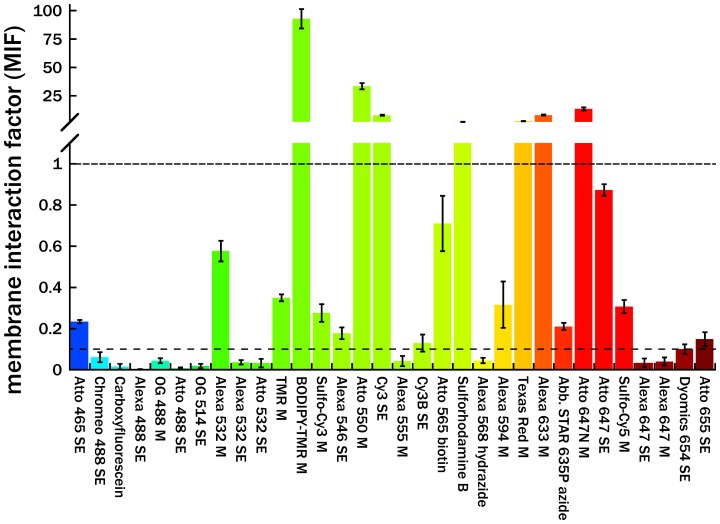
Bar graph of membrane interaction factors (MIF, **Table 1**), sorted by excitation maximum. Dyes below the bottom dashed line (MIF<0.1) exhibit little to no association with Egg PC lipid bilayers, while dyes above the second dashed line (MIF>1) strongly associate with membranes. Note that the y-axis changes substantially at MIF>1.1. Each data point represents the average of three independent measurements, ± propagated error of the standard deviation.

Despite being highly water-soluble, the 32 dyes we measured spanned nearly four orders of magnitude of MIF values (Figure 2 and Table 1). As a rough benchmark, we categorized the dyes in three groups. Dyes with MIF values <0.1 indicate very little membrane association. In our experience working with a subset of these dyes in fluorescence microscopy experiments, we have observed little or no evidence of membrane interaction. At the other extreme, our experience with dyes of MIF values >1 suggests they can interact strongly with membranes and introduce appreciable experimental artifacts. MIF values between these two extremes (0.1< MIF<1) indicate moderate levels of membrane-dye interaction, so we treat these dyes with caution. In general however, the tolerance for a given MIF value depends on the application and may be counterbalanced by other factors (spectral range, photophysics, etc.).

We observed several trends in our data. First, even though all the dyes are highly water-soluble, surprisingly few dyes had low MIF values (<0.1). Second, dyes excited with blue light (which tend to be smaller and more charged) generally had lower MIFs than redder dyes. However, there were exceptions to this trend (e.g. Alexa 647-M and Atto 465-SE). Third, as expected, highly charged dyes at physiological pH (net charge = −2 or −3) tended to have lower MIF values than uncharged or singly charged dyes. However, there were outliers, such as Alexa 633-M. Fourth, we observed some difference between reactive groups attached to the same dye (e.g. Alexa 532-M and Alexa 532-SE), but did not study this factor extensively. In our study, dyes were dissolved in buffer for 2 hours at room temperature to simulate common labeling reaction conditions, and then exposed to the vesicles. As a result, dyes with maleimides or succinimidyl esters likely contained some mixture of hydrolyzed and unhydrolyzed products when exposed to the vesicles, as would be the case for experimental protocols which label targets in a membranous environment. The hydrolysis of the maleimide or succinimidyl ester results in the introduction of an additional charged group which will likely decrease the propensity of the fluorophore to associate with the zwitterionic membrane.

**Figure 3:**
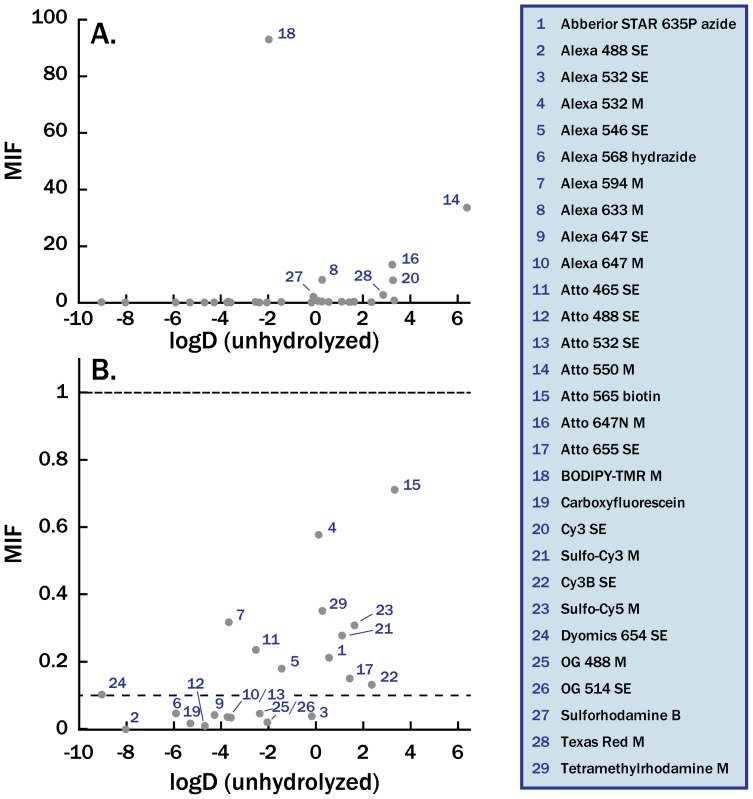
Correlation of MIF values with calculated log D. Calculated log D values are given in Table 1. (A) The MIF value shows moderate correlation with calculated log D values, based on the unhydrolyzed dye structures. (B) Zoom-in on dyes with MIF values below 1. Dyes with a MIF below the bottom dashed line (MIF<0.1) show little association with membranes, and dyes above the top dashed line (MIF>1) show appreciable interaction with lipid bilayers. MIF values shown here are the MIF_corr_ values given in Table 1.

After measuring the MIFs, we sought to determine whether the extent of membrane association could be predicted *a priori*. We calculated the distribution coefficient (log D, Table 1) for all dyes with published chemical structures (Figure S1). The log D values are calculations of the equilibrium partition coefficient of the dye molecule between octanol and water at pH 7.4, a common model system used to predict lipophilicity and pharamacokinetics. While there was a modest linear correlation between the log D coefficient and the measured MIF (r^2^ = 0.29 excluding BODIPY-TMR, see Figure 3), there were many outliers to this trend. In general, all dyes with log D>1 had MIF values in the intermediate (0.1<MIF<1) or substantial (MIF>1) range. Conversely, dyes with log D values<−4 tended to have low MIF values (≤0.1). For intermediate values (−4< logD<1), dyes with similar log D values had markedly different MIFs, such as Alexa 594-M and Atto 532-SE (MIF = 0.3 and log D = −3.66; MIF = 0.03 and log D = −3.58, respectively). Therefore, while log D values may be helpful in selecting a dye for a particular application, in our limited dataset they are not robust in predicting membrane interactions.

**Figure 4:**
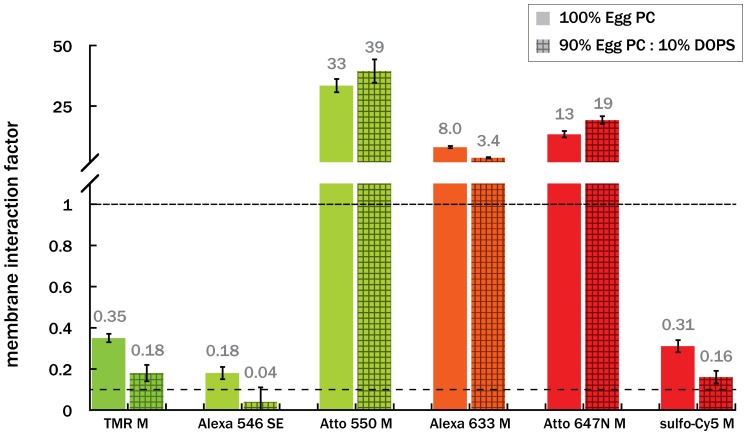
MIF_corr_neg_ values for a subset of dyes interacting with negatively charged 9∶1 Egg PC:DOPS vesicles. For comparison, the corrected MIF values of the dyes with pure Egg PC (MIF_corr_, solid bars) are shown to the left of the MIF values with DOPS (MIF_corr_neg,_ hashed bars). The MIF values in both lipid compositions, calculated as described in Equation 3, are displayed above each bar, and are given in Table 2. Dyes below the bottom dashed line (MIF<0.1) exhibit little to no association with Egg PC lipid bilayers, while dyes above the second dashed line (MIF>1) strongly associate with membranes. Note that the y-axis changes substantially at MIF>1.1. Each data point represents the average of three independent measurements, ± propagated error of the standard deviation. The difference between MIF_corr_neg_ and MIF_corr_ for all dyes shown is statistically significant at the p<0.05 level, as determined by a two-sample Kolmogrov-Smirnov test.

All the MIF values reported above were measured using vesicles with a zwitterionic lipid composition, Egg PC, which has been used to quantify drug-bilayer interactions [3]. For very different membrane compositions, especially those which are highly charged, the MIF value will likely change, perhaps even considerably. As an example of how the MIF values can shift with a charged lipid composition, we measured the MIF values for a subset of interesting dyes with Egg PC vesicles containing 10 mol% DOPS, a singly negatively charged lipid. We refer to MIF values for dyes measured against pure zwitterionic Egg PC as MIF_corr_ (Table 1), and dyes measured against 90% Egg PC:10% DOPS as MIF_corr_neg_ (Table 2). We chose six dyes with moderate or high MIF_corr_ values as test cases. We expected these dyes to be more affected by a negatively charged lipid composition than dyes with low MIF_corr_ values, which all contain multiple negative charges. As expected, the negatively charged dyes (Alexa 633-M, Alexa 546-SE, sulfo-Cy5-M, and TMR-M) had lower MIF_corr_neg_ values as compared to MIF_corr_ (Figure 4), whereas net neutral or positively charged dyes (Atto 550-M and Atto 647N-M) had higher MIF_corr_neg_ values. In the case of Alexa 633-M, which strongly associated with zwitterionic vesicles, MIF_corr_neg_ was less than MIF_corr_ but was still in the high range (MIF_corr_neg_ = 3.6), despite the dye having multiple negative charges. For dyes which showed moderate MIF_corr_ values (Alexa 546 SE, sulfo-Cy5-M, and TMR-M), MIF_corr_neg_ values were smaller than MIF_corr_, and in some cases small enough to be in the low MIF value regime (MIF<0.1). Clearly, the specific lipid composition can influence the association of a particular dye with the lipid bilayer, increasing or decreasing the interaction. For research applications with very different membrane compositions than those used herein, especially those which are highly charged, the MIF values should be re-measured using the method presented here or similar technologies [3], [8]. In principle, the method we present could even be extended to make measurements using cells in suspension, although careful attention would have to be paid to details such as concentration of cells to ensure sufficient signal-to-noise and incubation time to avoid artifacts from cellular death.

**Figure 5:**
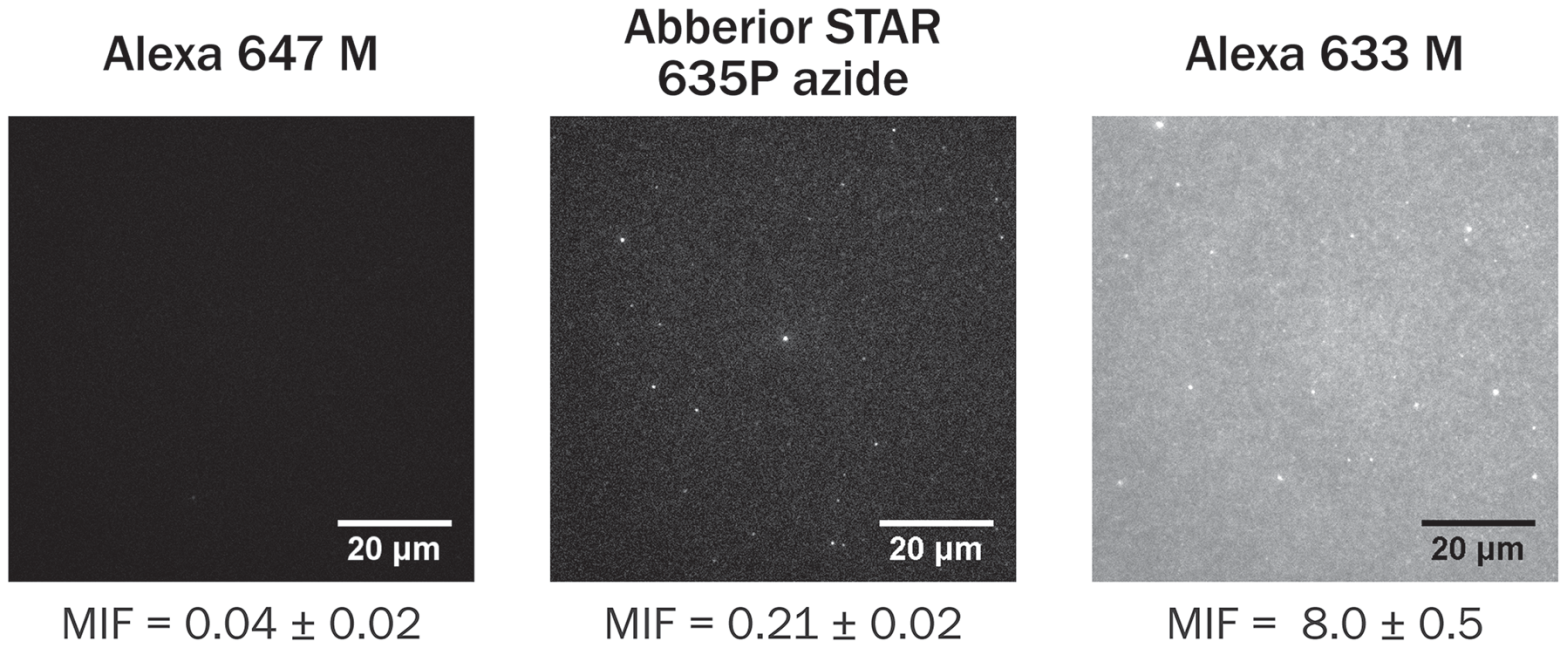
Observation of fluorophores interacting with lipid bilayers by fluorescence microscopy. After incubating a dye solution with a supported lipid bilayer and rinsing, any remaining fluorescence was imaged. The Alexa 647-M and Abberior STAR 635P azide images are set to the same contrast, while the Alexa 633-M sample was appreciably brighter.

Finally, to demonstrate the correlation between MIF values and the level of observable contamination in fluorescence microscopy experiments, we performed simple epifluorescence microscopy measurements on several dyes. After incubating a solution of a given dye above a glass-supported Egg PC lipid bilayer for 5 min, the bilayer was copiously rinsed with buffer, and any remaining fluorescence was observed (Figure 5). For dyes with low MIFs like Alexa 647-M, few dye molecules or aggregates were associated with the planar supported lipid bilayer after rinsing, while bilayers incubated with dyes with high MIFs like Alexa 633-M showed a high level of fluorescence. In many cases, these fluorophores appear to insert and diffuse along the plane of the bilayer, like a lipid-associated probe (Movie S1).

In summary, our data suggests that there is considerable variability in the level of membrane-dye interaction for common water-soluble fluorophores. While we observed general trends, there seemed to be no clear factor which could reliably predict the level of membrane-dye interaction for a given dye. Therefore, we recommend that a quantitative measure such as the MIF value be an important factor to consider when choosing a label for a particular experiment.

## Supporting Information

Figure S1 **Structures of the fluorescent dyes used in this study and their corresponding MIF values.** Chemical structures were obtained from the sources cited in Table 1. Note that the structure for Abberior STAR 635P azide is available upon request from Abberior. MIF values shown here are the MIF_corr_ values given in Table 1.
(TIF)

File S1 **File includes relationship between MIF value and equilibrium partition coefficient; Method rationale; Tables S1 and S2.**
(DOCX)

Movie S1 **After 10 nM Alexa 633-M is incubated with a glass-supported Egg PC bilayer for 5 minutes and rinsed thoroughly, Alexa 633-M particles remain associated with the bilayer and diffuse along the plane of the bilayer.**
(AVI)

Dataset S1 **Raw fluorescence spectra for all data used in this report.**
(XLSX)
