# Supplementary figures and images for: Choose Your Label Wisely: Water-Soluble Fluorophores Often Interact with Lipid Bilayers
Source: PLoS One. 2014 Feb 4;9(2):e87649. doi: 10.1371/journal.pone.0087649 (PMC3913624; doi:10.1371/journal.pone.0087649)

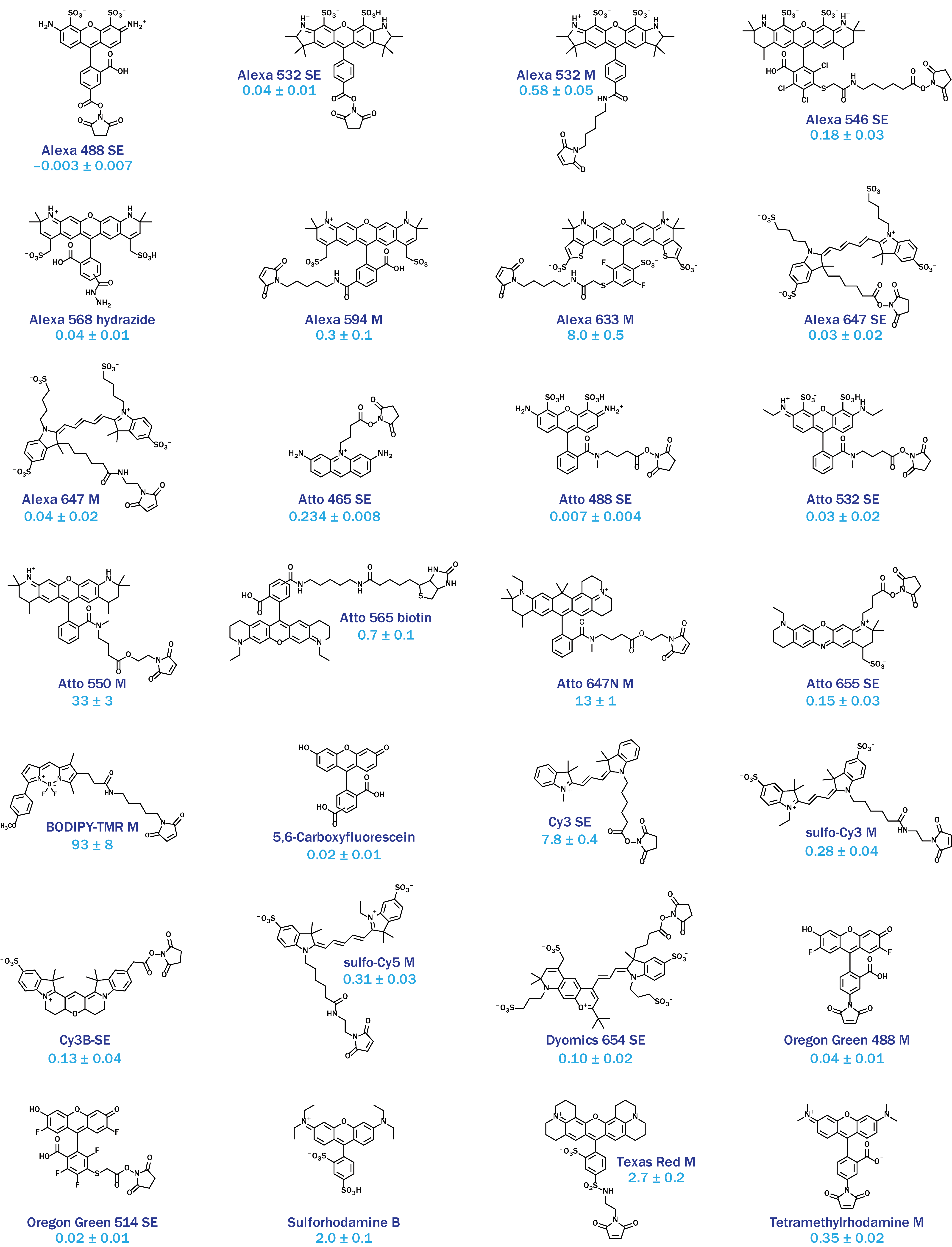

Supplement: Figure S1 — Structures of the fluorescent dyes used in this study and their corresponding MIF values. Chemical structures were obtained from the sources cited in Table 1. Note that the structure for Abberior STAR 635P azide is available upon request from Abberior. MIF values shown here are the MIFcorr values given in Table 1. (TIF) [file pone.0087649.s001.tif]
