# Supplementary material for: Choose Your Label Wisely: Water-Soluble Fluorophores Often Interact with Lipid Bilayers
Source: PLoS One. 2014 Feb 4;9(2):e87649. doi: 10.1371/journal.pone.0087649 (PMC3913624; doi:10.1371/journal.pone.0087649)
Supplement: File S1 — File includes relationship between MIF value and equilibrium partition coefficient; Method rationale; Tables S1 and S2. (DOCX) [file pone.0087649.s002.docx]

**Supporting Information**

**Choose your label wisely: water-soluble fluorophores often interact with lipid bilayers**

Laura D. Hughes^*^, Robert J. Rawle^*^, and Steven G. Boxer^‡^

^*^ These authors contributed equally.

^‡^ Corresponding author

# Relationship between MIF Value and the Equilibrium Partition Coefficient

The MIF value is related to the equilibrium partition coefficient and under appropriate circumstances will be the same to within a proportionality factor which is dependent on the lipid concentration used in the experiments (Eq. S1). The mole fraction equilibrium partition coefficient ($K_{x}$) can be calculated as

$K_{x}=\frac{\left(\frac{[dye]_{ves}}{[lipid]}\right)}{\left(\frac{[dye]_{H_{2}O}}{[H_{2}O]}\right)}$ Equation S1

where is $[dye]_{ves}$ the equilibrium concentration of dye that associates with the vesicles, $[dye]_{H_{2}O}$is the equilibrium concentration of dye in the buffer, and $[lipid]$ and $[H_{2}O]$ are the molar concentrations of lipid and water respectively in the experiment. Note that Eq. S1 is strictly accurate only in the limit of $[dye]_{ves}$<<$[lipid]$and $[dye]_{H_{2}O}$<<$[H_{2}O]$, as has been previously outlined by others [S1–S2]. Since $[dye]_{ves}$ and $[dye]_{H_{2}O}$ are proportional to the measured fluorescence $Fl_{ves}$ and $Fl_{H_{2}O}$ in our study, then $K_{x}$ is related to the MIF value as

$K_{x}=\frac{\left(\frac{[dye]_{ves}}{[lipid]}\right)}{\left(\frac{[dye]_{H_{2}O}}{[H_{2}O]}\right)}=\frac{\left(\frac{Fl_{ves}}{[lipid]}\right)}{\left(\frac{Fl_{H_{2}O}}{[H_{2}O]}\right)}=MIF\times\left(\frac{[H_{2}O]}{[lipid]}\right)$ Equation S2

For our system,([H_2_O]/[lipid]) is approximately 3.2x10^4^ and so in theory the MIF value could be converted to $K_{x}$ by multiplying by that factor. However, reporting $K_{x}$ requires several assumptions. Some of these assumptions are that 1) true equilibrium has been achieved, 2) the mechanism of interaction is known (for instance, if only the outer vesicle leaflet is accessible to the dye, then $[lipid]$must be divided by 2), and 3) the vesicle-dye interaction is not saturated for dyes with high MIF values at the concentrations used herein. Since we do not make those assumptions in the analysis of our data and since it is likely that the validity of those assumptions may vary depending on the dye, we chose to report the MIF values rather than the equilibrium partition coefficient and do not claim that the MIF is directly proportional to $K_{x}$. The MIF value is, however, sufficient to achieve the goal of this study, which is to identify and differentiate between dyes which have little, moderate, or considerable membrane interaction. For comparison purposes with other partition coefficients, we report a calculation of the equilibrium partition coefficient in Tables S1 and S2, but stress that this should be treated as a pseudo-equilibrium constant and so we denote it as$K_{x\_pseudo}$. We also note that the large ([H_2_O]/[lipid]) ratio—approximately 3.2x10^4^—means that even small measurement errors are greatly magnified when converting to $K_{x\_pseudo}$.

# Method Rationale

Several possibilities exist which could skew our calculated MIF values and yield false results. To rule out these possibilities, we designed our experimental method carefully and performed appropriate control experiments. Each is outlined below.

One possibility is that the fluorescence properties (quantum yield, emission maximum, etc.) of some dyes may be altered as they interact with the vesicles. This could yield spurious results. To address that possibility, all samples were mixed with 1% Triton-X detergent immediately before fluorescence measurement following the 3 day dialysis (see Methods in the Main Text). This concentration of Triton-X is approximately 13 times the lipid concentration in vesicle samples and will readily lyse all vesicles—eliminating potential light scattering—creating a nearly identical environment for dyes whether in the presence of lysed vesicles or not. To test this, a control experiment was performed using Atto 647N-M, which we previously observed to have an appreciable shift in its fluorescence properties upon vesicle interaction. Two solutions of Atto 647N-M were prepared at the same dye concentration, vesicles were added to one solution, and both solutions were mixed with 1% Triton-X in PBS and then fluorescence spectra were obtained. The resulting spectra for both samples were observed to be equivalent (data not shown), verifying our usage of 1% Triton-X to negate any changes in fluorescence properties in the dye upon interaction with the vesicles.

Another possibility is that some of the dyes (especially those with high MIF values) are also likely to stick to the dialysis membrane, microcentrifuge tube, etc., and so the final dye concentration in solution may be considerably less than what was originally added. Furthermore, the original measurement of dye concentration, measured by absorption at λ_ex,max_, may be skewed by inaccurate published extinction coefficients, which are often obtained in different solvents than PBS. However, the MIF value is ratiometric and does not depend on the actual concentration of dye and so this possibility is not an issue.

A third possibility is that anti-cooperative dye-membrane interactions may yield underestimates for dyes with high MIF values. For example, if many dyes with a single charge bind to a lipid vesicle, this could prevent binding of additional dyes because the accumulated charges on the vesicle would repel further binding. This possibility is likely an issue for some of the dyes with high MIF values >1, but since a high MIF value already demonstrates considerable membrane interaction, we are less concerned that some of those values may be underestimates.

A fourth possibility is that the dialysis dye solution may contain a mixture of hydrolyzed and unhydrolyzed products for those dyes with hydrolysable reactive groups such as maleimides or succinimidyl esters. The dye solutions were prepared in PBS buffer for 2 h before exposing them to the vesicle solution in the dialysis cassette and some amount of hydrolysis likely occurred during this 2 h incubation. If there is a propensity for one product to interact more strongly with the lipid vesicles, then this could skew the apparent MIF value, particularly as the hydrolysis of maleimides or succinimidyl esters would introduce an additional charged group at physiological pH. This possibility is likely an issue for many of the dyes we studied, although it is unclear to what extent hydrolysis would shift the measured MIF value. However, we do note that our experimental conditions were chosen to mimic common labeling procedures and therefore we think the MIF measurement reported herein is actually the more relevant measurement to guide in dye selection because it captures some of the messiness of experimental labeling protocols. This possibility is, however, another reason why assuming that the MIF value is directly proportional to $K_{x}$ may be treacherous—the exact mixture of hydrolyzed and unhydrolyzed products is not known.

# Supporting References

S1. White SH, Wimley WC, Ladokhin AS, Hristova K (1998) [4] Protein folding in membranes: Determining energetics of peptide-bilayer interactions. Methods Enzymol. 295: 62-87.

S2. Ladokhin AS, Selsted ME, White SH (1997) Bilayer Interactions of Indolicidin, a Small Antimicrobial Peptide Rich in Tryptophan, Proline, and Basic Amino Acids. Biophys. J. 72: 794-805.

# Supporting Tables

**Table S1.** Corrected and Raw MIF Values and Pseudo Equilibrium Partition Constants for Common Water Soluble Dyes, Measured Against Zwitterionic Vesicles (100% Egg PC)

| **Dye^a^** | **MIF_corr_^b^** | **MIF_raw_^c^** | **Kx_pseudo^d^** |
| --- | --- | --- | --- |
| Abberior STAR 635P azide | 0.21 ± 0.02 | 0.053 ± 0.004 | 6.7E+3 ± 5E+2 |
| Alexa 488 SE | -0.003 ± 0.007 | 0.001 ± 0.004 | -1E+2 ± 2E+2 |
| Alexa 532 SE* | 0.04 ± 0.01 | 0.032 ± 0.007 | 1.2E+3 ± 4E+2 |
| Alexa 532 M | 0.58 ± 0.05 | 0.5 ± 0.1 | 1.8E+4 ± 2E+3 |
| Alexa 546 SE | 0.18 ± 0.03 | 0.15 ± 0.02 | 5.7E+3 ± 9E+2 |
| Alexa 555 M | 0.04 ± 0.03 | 0.05 ± 0.02 | 1.3E+3 ± 8E+2 |
| Alexa 568 hydrazide | 0.04 ± 0.01 | 0.06 ± 0.01 | 1.4E+3 ± 4E+2 |
| Alexa 594 M | 0.3 ± 0.1 | 0.18 ± 0.1 | 1.0E+4 ± 4E+3 |
| Alexa 633 M | 8.0 ± 0.5 | 7.7 ± 0.4 | 2.6E+5 ± 1E+4 |
| Alexa 647 SE | 0.03 ± 0.02 | 0.02 ± 0.01 | 1.1E+3 ± 7E+2 |
| Alexa 647 M | 0.04 ± 0.02 | 0.047 ± 0.003 | 1.3E+3 ± 6E+2 |
| Atto 465 SE | 0.234 ± 0.008 | 0.19 ± 0.01 | 7.5E+3 ± 2E+2 |
| Atto 488 SE | 0.007 ± 0.004 | 0.003 ± 0.004 | 2E+2 ± 1E+2 |
| Atto 532 SE | 0.03 ± 0.02 | 0.02 ± 0.02 | 1.0E+3 ± 7E+2 |
| Atto 550 M** | 33 ± 3 | 26 ± 0.8 | 1.07E+6 ± 9E+4 |
| Atto 565 biotin | 0.7 ± 0.1 | 0.7 ± 0.1 | 2.3E+4 ± 4E+3 |
| Atto 647 SE** | 0.87 ± 0.03 | 0.87 ± 0.02 | 2.80E+4 ± 9E+2 |
| Atto 647N M | 13 ± 1 | 10.7 ± 0.2 | 4.3E+5 ± 4E+4 |
| Atto 655 SE | 0.15 ± 0.03 | 0.14 ± 0.02 | 5E+3 ± 1E+3 |
| BODIPY-TMR M | 93 ± 8 | 91 ± 8 | 3.0E+6 ± 3E+5 |
| Carboxyfluorescein | 0.02 ± 0.01 | 0.01 ± 0.01 | 5E+2 ± 4E+2 |
| Chromeo 488 SE | 0.06 ± 0.02 | 0.05 ± 0.02 | 2.0E+3 ± 8E+2 |
| Cy3 SE | 7.8 ± 0.4 | 6.3 ± 0.3 | 2.5E+5 ± 1E+4 |
| sulfo-Cy3 M | 0.28 ± 0.04 | 0.28 ± 0.04 | 9E+3 ± 1E+3 |
| Cy3B SE | 0.13 ± 0.04 | 0.12 ± 0.04 | 4E+3 ± 1E+3 |
| sulfo-Cy5 M | 0.31 ± 0.03 | 0.27 ± 0.03 | 9.8E+3 ± 1E+3 |
| Dyomics 654 SE | 0.10 ± 0.02 | 0.09 ± 0.01 | 3.2E+3 ± 8E+2 |
| OG 488 M | 0.04 ± 0.01 | 0.03 ± 0.01 | 1.4E+3 ± 4E+2 |
| OG 514 SE | 0.02 ± 0.01 | 0.04 ± 0.01 | 6E+2 ± 3E+2 |
| Sulforhodamine B | 2.0 ± 0.1 | 1.86 ± 0.06 | 6.4E+4 ± 3E+3 |
| Texas Red M | 2.7 ± 0.2 | 2.5 ± 0.1 | 8.5E+4 ± 6E+3 |
| TMR M | 0.35 ± 0.02 | 0.31 ± 0.02 | 1.12E+4 ± 5E+2 |

^a^ Reactive groups include maleimides (M), azide, biotin, hydrazide, and succinimidyl esters (SE). Where available, dye structures are given in Figure S1.

^b^ Corrected MIF value, measured using zwitterionic vesicles (100% Egg PC) and calculated using Equation 3 in Main Text. Error values are the propagated error from the standard deviation of three separate measurements each of the experimental and control samples. These are the same values as in Table 1 of Main Text.

^c^ Raw MIF value, measured using zwitterionic vesicles (100% Egg PC) and calculated using Eq. S2 and averaged across three separate measurements. Error values are the standard deviation of three separate measurements.

^d^ The pseudo equilibrium partition coefficient. Calculated using Equation S2, where MIF = MIF_corr_ and using [H_2_O] = 55.5M and [lipid] = 1.73 mM (estimated from nominal starting lipid concentration before vesicle extrusion). Error values are the propagated error from the standard deviation of three separate measurements each of the experimental and control samples.

**Table S2.** Corrected and Raw MIF Values and Pseudo Equilibrium Partition Constants for Common Water Soluble Dyes, Measured Against Negatively Charged Vesicles (90% Egg PC, 10% DOPS)

| **Dye^a^** | **MIFcorr_neg^b^** | **MIFraw_neg^c^** | **Kx_pseudo^d^** |
| --- | --- | --- | --- |
| Alexa 546 SE | 0.04 ± 0.07 | 0.05 ± 0.03 | 1E+3 ± 2E+3 |
| Alexa 633 M | 3.6 ± 0.3 | 3.5 ± 0.2 | 1.17E+5 ± 9E+3 |
| Atto 550 M | 39 ± 5 | 35 ± 2 | 1.3E+6 ± 2E+5 |
| Atto 647N M | 19 ± 2 | 19 ± 2 | 6.2E+5 ± 5E+4 |
| sulfo-Cy5 M | 0.16 ± 0.03 | 0.130 ± 0.003 | 5E+3 ± 1E+3 |
| TMR M | 0.18 ± 0.04 | 0.15 ± 0.02 | 6E+3 ± 1E+3 |

^a^ Reactive groups include maleimides (M), azide, biotin, hydrazide, and succinimidyl esters (SE). Where available, dye structures are given in Figure S1.

^b^ Corrected MIF value, measured using negatively charged vesicles (90% Egg PC, 10% DOPS), and calculated using Equation 3 in Main Text. Error values are the propagated error from the standard deviation of three separate measurements each of the experimental and control samples. These are the same values as in Table 1 of Main Text.

^c^ Raw MIF value, measured using negatively charged vesicles (90% Egg PC, 10% DOPS), calculated using Equation S2 and averaged across three separate measurements. Error values are the standard deviation of three separate measurements.

^d^ The pseudo equilibrium partition coefficient. Calculated using Equation S2, where MIF = MIFcorr_neg and using [H_2_O] = 55.5M and [lipid] = 1.72 mM (estimated from nominal starting lipid concentration before vesicle extrusion). Error values are the propagated error from the standard deviation of three separate measurements each of the experimental and control samples.
